# Challenging the holy grail of hospital accreditation: A cross sectional study of inpatient satisfaction in the field of cardiology

**DOI:** 10.1186/1472-6963-10-120

**Published:** 2010-05-12

**Authors:** Cornelia Sack, Peter Lütkes, Wolfram Günther, Raimund Erbel, Karl-Heinz Jöckel, Gerald J Holtmann

**Affiliations:** 1 Department of Strategy and Medical Planning, University Hospital Essen, Germany; 2 Department of Medical Controlling, University Hospital Essen, Germany; 3 Picker Institute Germany, Germany; 4 Clinic for Cardiology, University Hospital Essen, Germany; 5 Institute for Medical Informatics, Biometry and Epidemiology, University Hospital Essen, Germany; 6 University Hospital Essen, Executive Board, Germany; 7 University of Adelaide, Faculty of Health Sciences, Australia

## Abstract

**Background:**

Subjective parameters such as quality of life or patient satisfaction gain importance as outcome parameters and benchmarks in health care. In many countries hospitals are now undergoing accreditation as mandatory or voluntary measures. It is believed but unproven that accreditations positively influence quality of care and patient satisfaction. The present study aims to assess in a defined specialty (cardiology) the relationship between patient satisfaction (as measured by the recommendation rate) and accreditation status.

**Methods:**

Consecutive patients discharged from 25 cardiology units received a validated patient satisfaction questionnaire. Data from 3,037 patients (response rate > 55%) became available for analysis. Recommendation rate was used as primary endpoint. Different control variables such as staffing level were considered.

**Results:**

The 15 accredited units did not differ significantly from the 10 non-accredited units regarding main hospital (i.e. staffing levels, no. of beds) and patient (age, gender) characteristics. The primary endpoint "recommendation rate of a given hospital" for accredited hospitals (65.6%, 95% Confidence Interval (CI) 63.4 - 67.8%) and hospitals without accreditation (65.8%, 95% CI 63.1 - 68.5%) was not significantly different.

**Conclusion:**

Our results support the notion that - at least in the field of cardiology - successful accreditation is not linked with measurable better quality of care as perceived by the patient and reflected by the recommendation rate of a given institution. Hospital accreditation may represent a step towards quality management, but does not seem to improve overall patient satisfaction.

## Background

A key parameter that is believed to measure quality of care in a hospital setting is patient satisfaction [1-3]. Patient satisfaction is as equally important as traditional outcome parameters such as mortality or functional status. As a result health care organizations perceive patient satisfaction as an important factor that plays a pivotal role in a competitive health care market.

Patient satisfaction can be measured utilizing standardized questionnaires or interviews [4]. While there are published data comparing patient satisfaction of different health care providers, doctors and hospital managers operating in a competitive market are constantly pressured to implement changes to improve patient satisfaction. A number of factors are believed to influence patient satisfaction including staffing levels, infrastructure, or discharge information [5-7], which are also important topics in the accreditation process.

In recent decades there is an emerging trend that accreditation or certification is a feasible measure to improve the quality of care and patient safety [8]. The terms 'Accreditation' and 'certification' are interchangeable; 'accreditation' is used in the USA, while 'certification' is used in Germany. For the interest of this study, the term accreditation will be used. Many hospitals have their own internal quality assurance, but also aim to meet specific external standards with regard to the standardization of clinical measures and clinical pathways. In many countries external evaluation by independent assessors resulting in accreditation are measures that are believed to improve quality of care [9]. Indeed, in many countries hospital accreditation is voluntary or a legal requirement. In the USA, for example, it is a requirement for hospitals to have accreditation to become providers in the Medicare program [10]. In France accreditation has been compulsory since 1996 [11]. In Germany accreditation is voluntary while quality assurance (comparison of key outcome data) is mandatory in many disciplines. Many perceive accreditation as a measure to improve quality of care or to document superiority of the quality of care, therefore many hospitals aim for accreditation to achieve a competitive advantage [12]. The patient's perspective is being incorporated into almost every quality measure as hospitals recognize patient satisfaction as an important outcome of health service [13]. Because of higher competition and public reporting of hospital quality in the USA and other countries, healthcare practitioners are being scrutinized and compared with external standards. In this context, accreditation is believed to be, at least, an indicator for reasonable quality (or even superior quality of care if accreditation is voluntary) that can be easily identified by patients and referring doctors. If an organization does not go through an accreditation process, it may indicate that the facility is not open to external evaluation of its performance and may lead to competitive disadvantage [14]. Health care planners, regulators and payers are strongly supporting accreditation while physicians are more hesitant since accreditation is frequently perceived as a bureaucratic exercise that distracts from the core activities. Appropriate accreditation is an arduous process that requires substantial resources, however studies investigating its beneficial effects regarding key outcome parameters are widely lacking [15]. As stated above, the factors on which patient satisfaction are influenced correspond widely to the topics on which accreditation processes are based on. Patient satisfaction is often a main topic in the accreditation process, as patient satisfaction is dependent on the perceived quality of information, communication and organization [14]. These topics are also important to succeed in the accreditation process [14].

A number of previous studies have investigated the benefits of accreditation of health care providers [16], and describe the different accreditation standards. Many papers have addressed accreditation standards which are similar to American or Australian standardization. In addition, some researchers indicate that the accreditation improves their operations and performance in terms of effectiveness and efficiency [16]. All accreditation efforts require resources and regarding the aim of evidence based management the rationale for allocation of resources and the return of investment should be measured. Interestingly, data quantifying the economical and quality effect of accreditation are widely lacking. As emphasised by Greenfield & Braithwaite [15] there are limited data on the influence of accreditation of health care providers on patient satisfaction. The existing studies [17,18] have major limitations, i.e. small sample sizes or not administering validated instruments to assess patient satisfaction. However, these studies did not find an association between patient satisfaction and hospital accreditation. Other studies looking at performance measures like Patient Safety Indicators or survival rates on special diseases and accreditation did not find any or no clear relationships between them as well [19,20]. Whether accrediting of hospitals is truly ensuring high quality healthcare is a question that remains to be answered, which indicates there is the need to provide evidence that accreditation procedures indeed result in improved patient satisfaction.

The present study aims to assess in a defined speciality (cardiology) the relationship between patient satisfaction (as measured by the recommendation rate) and accreditation status. We hypothesized that accredited hospitals have higher patient satisfaction when compared with non-accredited hospitals.

## Methods

A widely accepted accreditation system designed for hospitals in Germany is KTQ (Cooperation for Transparency and Quality in Hospitals) [14]. An alternative model is proCum Cert (pCC). Both models are very similar to the JCAHO (Joint Commission on Accreditation of Healthcare Organizations) accreditation standards which mean that a hospital undergoes an independent review of the organization's performance against national quality and safety requirements. They aim towards an organization wide accreditation. The KTQ- and pCC-accreditation processes consist of a self assessment followed by an external assessment. Both procedures are based on a nearly identical criteria-catalogue [14].

In 2007 a regional patient survey was conducted involving 75 hospitals in the Ruhr area, comprising of more than 50% of all hospitals in a highly urbanized area with greater than six million inhabitants. The Picker questionnaire was mailed to 78,508 patients [21,22] between two and eight weeks after their discharge. Patients who did not answer the questionnaire were sent a first, and if necessary a second reminder four weeks after the last letter. A total of 44,418 (57%) responded. For our analysis we focused on patients discharged from dedicated departments of cardiology in order to decrease variability due to a large number of different diagnoses and procedures. These departments could be identified because the discharging unit was mentioned on the Picker questionnaire. In our study, accreditation was defined as the hospitals having received approval and received a certificate. Nine out of 25 cardiology departments were certified according to KTQ, six according to both KTQ and pCC. The remaining ten departments were not accredited. 1,835 patients were treated in an accredited unit, whereas 1,202 patients were treated in non-accredited units. Two departments were excluded because of an accreditation system that is not comparable to KTQ and pCC. We included patients treated in one of 25 specialized cardiology departments leaving a sample size of 3,037 patients.

The Picker Inpatient Questionnaire assesses seven dimensions of patient satisfaction [23]. Jenkinson et al. demonstrated in a study over five countries that the problem scores for many of the Picker-Items are often high (>20%), but the study also indicated that the rates are not comparable between different hospitals and countries [24]. However, a parameter that reflects overall satisfaction is the 'recommendation rate' of a specific health care provider [24]. This is the proportion of patients that would recommend a specific institution based upon their experiences. This parameter was used as primary outcome parameter.

The following study is based on a Picker Questionnaire and Picker factors that were developed for the special needs of hospitals in Germany. The German factors differ from the Picker dimensions in Jenkinson et al. [23].

All individual questions that measured potential problems were dichotomized, that is, i.e. were assigned to one of two groups depending on whether a problem was mentioned [24]. A problem score for each of the ten Picker factors was calculated on the basis of this problem rating. The problem score reflects the percentage of responses to questions that indicated a problem with care. Higher scores indicate more problems. The mean problem scores for factors of care were compared between the hospitals.

**Figure 1:**
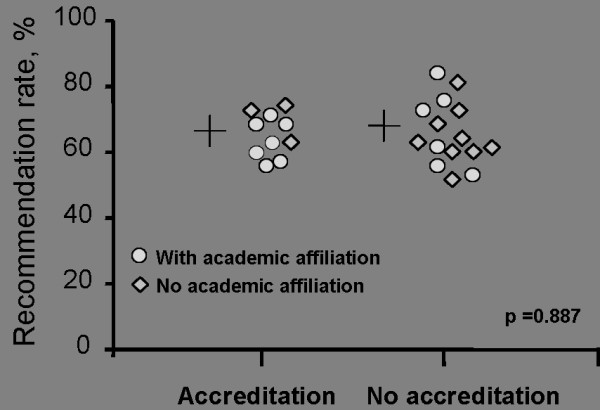
**Comparison of recommendation rate for accredited and non-accredited hospitals**. The O depicts departments with academic affiliation (teaching hospital) whereas ◊ reflects hospitals without academic affiliation. The mean recommendation rate of the groups accredited vs. not accredited is not significant (Chi Square Test, p = 0,887).

Mean values and STD or percentages with 95% confidence intervals (CI) are reported where appropriate. All p values calculated were two-tailed; non parametric tests were used where appropriate. A p-value < 0.05 was considered significant for the primary parameters with Bonferroni's correction to control for type I error for the secondary outcome parameters. Different control variables, such as staffing level were analysed between the two groups (accredited vs. non-accredited) (tables 1 and 2). Mann Whitney U test was used to compare descriptive statistics for patients in accredited and non-accredited hospitals (table 1) and to compare hospital characteristics (table 2). Mean problem scores by factors of the Picker questionnaire for accredited and non-accredited hospitals were compared using Mann Whitney U test (table 3). Patients' overall assessment of the quality of care for accredited and non-accredited hospitals was analysed using Chi Square test and Mann Whitney U test (table 4). Figure 1 shows the recommendation rate for every hospital subdivided in hospitals with and without academic affiliation and in hospitals with and without accreditation. Using Chi Square test we compared the mean recommendation rates for these groups.

**Table 1:** Descriptive statistics for patients in accredited and non-accredited hospitals*, data from 25 hospitals

	accredited		non-accredited		P
Age	1735	68.1 (11.9)	1153	67.7 (12.4)	0.484^u^
Length of stay	1685	9.7 (8.7)	1081	10.6 (10.5)	0.104^u^
Gender					0.177^c^
Female	726 (41.3%)		448 (38.8%)		0.538^c^
Nationality of patients					
German	1597 (96.5%)		1094 (96.0%)		
migrants	58 (3.5%)		45 (4.0%)		

*Note. Cells include either the mean +/- standard deviation or cell size and column percentages.

^c ^Chi Square test (Pearson)

^u ^Mann Whitney U test

**Table 2:** Descriptive statistics for accredited and non-accredited hospitals*, data from 25 hospitals, STD = standard deviation

	accredited		non-accredited		P
	n	Mean (STD)	n	Mean (STD)	Value
Teaching hospital					0.141^c^
Yes		6 (40.0%)		7 (70.0%)	
No		9 (60.0%)		3 (30.0%)	
Ownerstatus					0.071^c^
Public		0 (0%)		2 (20.0%)	
Non-profit		15 (100.0%)		8 (80.0%)	
No of beds	13	60 (22)	9	69 (29)	0.482^u^
Case load	12	3353 (2089)	7	3489 (2078)	0.933^u^
Physician/bed ratio	13	0.25 (0.07)	9	0.26 (0.08)	0.616^u^
Specialist/bed ratio	15	0.41 (0.14)	10	0.48 (0.08)	0.141^u^
Nurse/bed ratio	13	0.66 (0.15)	9	0.89 (0.65)	0.548^u^
Proportion RNs	15	0.83 (0.08)	10	0.87 (0.07)	0.166^u^

*Note. Cells include either the mean +/- standard deviation or cell size and column percentages.

^u^Mann Whitney U test

^c^Chi Square test (Pearson)

**Table 3:** Problem scores by factors of the Picker questionnaire for accredited and non-accredited hospitals, data from 25 hospitals, STD = standard deviation

	accredited		non-accredited		p
Picker factors	n	Mean (STD)	n	Mean (STD)	**Value**^**u**^
Physician-patient relation	1825	18.09 (27.57)	1196	17.11 (26.40)	0.538
Nurse-patient relation	1825	14.85 (25.56)	1199	15.19 (25.12)	0.341
Treatment success	1791	17.52 (30.60)	1182	18.63 (31.10)	0.232
Quality of accomodation	1749	26.30 (36.24)	1156	24.22 (35.32)	0.122
Quality of catering	1754	15.04 (25.52)	1150	16.30 (26.14)	0.136
Admission procedure	1781	22.94 (37.14)	1176	25.13 (39.14)	0.232
Discharge procedure	1647	51.08 (42.44)	1067	51.55 (42.19)	0.797
Perceived cleanliness	1780	11.46 (29.39)	1160	11.38 (29.08)	0.940
Inclusion of relatives/friends	1101	30.61 (40.10)	757	32.03 (41.08)	0.527
Room atmosphere	1290	24.57 (36.44)	863	20.95 (33.58)	0.055

^u^Mann Whitney U test

**Table 4:** Patients' overall assessment of the quality of care for accredited and non-accredited hospitals*, data from 25 hospital, STD = standard deviation

	accredited		non-accredited		P
	n	Mean (STD)	n	Mean (STD)	Value
Cooperation physicians and nursing staff	1775	3.31 (0.81)^1^	1159	3.31 (0.80)^1^	0.629^u^
Perceived quality of care	1792	3.41 (0.84)^1^	1175	3.39 (0.83)^1^	0.355^u^
Judgment of length of stay	1774	3.00 (0.57)^2^	1157	2.98 (0.59)^2^	0.623^u^
Recommendation rate*:					0.887^c^
Definitively yes	1171 (65.6%)		776 (65.8%)		
All other resonses	615 (34.4%)		403 (34.2%)		

^u^Mann Whitney U test

^c^Chi Square test (Pearson)

*Note. Cell includes cell size and column percentages

^1^Scale from 1 to 5: 1 = poor to 5 = excellent

^2^Scale from 1 to 5: 1 = much shorter than necessary to 5 = much longer than necessary

As the survey was anonymous and the questionnaires were sent to the Picker Institute and not to the hospital, an ethical approval was not necessary. All statistical analyses were made with the Statistical Package for the Social Sciences (SPSS Inc., Chicago, Illinois, USA, Release 13.0, 2004).

## Results

The key characteristics of accredited and non-accredited hospitals are depicted in tables 1-2. There were no significant differences between accredited and non-accredited hospitals for length of stay, gender, age or the proportion of foreign patients. However, there was a trend that accredited hospitals were more likely to be non-profit and not affiliated teaching hospitals (p < 0.15).

### Recommendation rate

Rates of institutions with accreditations (65.6%, 95% CI 63.4 - 67.8%) were not significantly different from institutions without formal accreditation (65.8%, 95% CI 63.1 - 68.5%, Figure 1).

### Specific factors of patient satisfaction

With respect to the factor specific problem scores, key factors of patient satisfaction indicated no significant advantage for accredited units (table 3). We found no significant difference between the two hospital groups regarding the physician and nursing staff as perceived by patients or regarding satisfaction with the admission or discharge procedures. Overall quality ratings for cooperation of medical and nursing staff as perceived by patients, overall evaluation of care and assessment of duration of stay were not significantly different (table 4) between the two hospital groups.

## Discussion

The main finding of this cross sectional study which included more than 3,000 patients discharged from cardiology units from 25 different hospitals after inpatient treatment was that there is no significant difference for the recommendation rate between accredited (65.6%, 95% CI 63.4 - 67.8%) and non-accredited (65.8%, 95% CI 63.1 - 68.5%) hospitals. While it might be argued that any advantage of accredited hospitals might be simply missed due to a type II error, it is important to note that the recommendation rates of some hospitals were numerically higher in non-accredited units. In addition, more than 3,000 patients were included into this study and we made a purposeful selection of patients by specialty, therefore it is highly unlikely that the somehow surprising result is due to insufficient sample size.

A recommendation rate above 60% is considered high, however, there is still substantial room for improvement. Thus a ceiling effect that probably makes it difficult to identify differences can be excluded as a potential cause for the lack of a difference. In addition, we assessed numerous other secondary outcome parameters such as perceived quality of admission or discharge procedures. Similar to the primary outcome parameter, there were no significant differences. The only exception was the quality rating for the room atmosphere with a trend in favour of non-accredited institutions.

Accreditation procedures are believed to be suitable measures to improve quality of care including patient satisfaction. But our results indicate that hospital accreditation process does not correlate with patient satisfaction and service quality as perceived by the patient, therefore providing evidence that an accreditation is not linked to improved patient satisfaction.

As stated above, there are many studies which have investigated the benefits of accreditation of health care providers [16,25-27]. Accreditation may be advantageous regarding standardization of procedures, cost containment or marketing. However, there are reservations that accreditation is a suitable instrument for quality improvements that are relevant to patient satisfaction. Hospitals are accredited for their compliance with standards [28,29]. Indeed, accreditation programs focus primarily on structures and processes in patient care, e.g. the patients and their way from admission to discharge [30]. The underlying assumption is that if hospital pathways and processes are properly regulated and controlled, patient outcomes and patient satisfaction are likely to be improved. To increase outcome orientation in the accreditation process, there is a need for quality indicators such as morbidity adjusted mortality and patient satisfaction. But until now, there are only few examples of performance measures in the accreditation process standards [30].

While cost containment in hospitals is an issue in many countries, there is an obvious need to correctly establish costs and benefits of accreditation. The process of accreditation requires resources and time [16,31]. For example, Staines [31] describes the process of a ISO accreditation of a small Swiss hospital, required three years of work from three full time staff members. This figure may reflect only a small proportion of the true costs of an accreditation process, as implementation also requires substantial input from a large number of other stakeholders, such as doctors and nursing staff. For an accreditation program to be cost effective the gain needs to be measurable and improvement of patient satisfaction is an important outcome parameter. Considering this, it is surprising that until now there is limited data that quantify the influence of accreditation on patient satisfaction.

It is important to note that our study also has some limitations which need to be considered but are unlikely to explain the lack of an association between patient satisfaction and accreditation. Firstly, accreditation is based on a continuous quality improvement process. For this reason, it is hard to define the endpoints of accreditation so that changes in process and outcome measures may develop over time. In addition, many hospitals in our study had just received their certificate, thus they have taken significant steps within the accreditation process, but the full benefits may emerge at a later time point. We do not know exactly when the hospitals received their certificates, but we assume that it was recently as accreditation of hospitals was only introduced to Germany in 2008. This may explain why, in our study, some non-accredited institutions were numerically better for recommendation rates, and it is unlikely that the time effect will change the results. Secondly, it is possible that some hospitals were given provisional accreditation with recommendations for improvement. If so, hospitals may have been accredited despite their existing problems. Thirdly, individual accreditation programs vary with respect to scope and standards and we did not consider these differences between the two accreditation programs. However, considering the large sample size it is unlikely that any relevant association would have been missed. In addition, it could be argued that hospitals with accreditation would be worse if they did not undergo the accreditation process. However, there is no evidence (e.g. based upon the change of case load in the past) to support this assumption.

## Conclusions

In summary, this study including more than 3,000 patients discharged from 25 cardiology units after inpatient treatment does not demonstrate any significant association between hospital accreditation and the primary or secondary outcome parameters reflecting patient satisfaction (recommendation rate and satisfaction with care). Hospital managers frequently see accreditation as a means to maintain high standards in health care delivery. However, our data may suggest that at least some of the currently established accreditations fail to improve patient satisfaction. This can be considered an important issue and further research is needed to identify the factors that determine patient satisfaction that can be effectively targeted. In addition, our data may lead towards the need to properly assess the effects of accreditation to provide data that are needed for evidence based decision making when it comes to future improvements of accreditation in health care.

Perhaps, the focus of our study on cardiology units leaves the question if results could be replicated or if they would differ if the study was conducted in patients within another medical discipline. This question is an important subject for future research.

## Competing interests

The authors declare that they have no competing interests.

## Authors' contributions

CS contributed to the drafting of the paper, performed statistical analysis, analysed and interpreted data and participated in the critical revision of the manuscript for important intellectual content. PL analysed and interpreted data and participated in the critical revision of the manuscript for important intellectual content. WG participated in the data acquisition and in the critical revision of the manuscript for important intellectual content. RE participated in the conception and design and in the critical revision of the manuscript for important intellectual content. KJ participated in the conception and design and in the critical revision of the manuscript for important intellectual content. GH had the idea for this study contributed to paper drafting, performed statistical analysis, analysed and interpreted data and participated in the critical revision of the manuscript for important intellectual content. All authors read and approved the final manuscript.

## Pre-publication history

The pre-publication history for this paper can be accessed here:

http://www.biomedcentral.com/1472-6963/10/120/prepub
